# Factors Influencing Mental Health Literacy in Indian Tribal Communities

**DOI:** 10.1192/bjo.2025.10092

**Published:** 2025-06-20

**Authors:** Ehsas Kakkar, Paramita Bhattacharya, Nirmalaya Mukherjee, Subhash Pokhrel

**Affiliations:** ^1^Brunel Medical School, Uxbridge, United Kingdom; ^2^MANT (Manbhum Ananda Ashram Nityananda) Organisation, Kolkata, India; ^3^Brunel University, Uxbridge, United Kingdom

## Abstract

**Aims:** About 9% of India’s population is made up of tribal communities consisting 120 million people nationwide. The mental health literacy of these communities is an unexplored topic. This literature review is aimed to identify and summarise existing factors that influence mental health literacy of the tribal communities in India and use this information to develop a survey questionnaire to support the development of a community radio programme.

**Methods:** SCOPUS, PubMed, Cochrane Library, Web of Science Journal, and Google Scholar were searched for peer-reviewed articles published in English from 1991–2024. Qualitative, quantitative, and mixed methods studies focusing on mental health knowledge and awareness reported from India were selected following PRISMA. Papers were appraised using the Calgary Health Region critical appraisal tool and extracted data were analysed using a narrative synthesis. The key findings were used to develop a survey questionnaire to support the development of a community radio programme to promote mental health literacy among the Indian tribal population.

**Results:** Out of 103 papers initially identified, 11 papers were chosen for full text review, data extraction, and analysis upon critical appraisal. Cultural differences, access to healthcare, economic status and lack of education were found to be key factors influencing mental health in tribal communities, through the investigation.

**Conclusion:** The corresponding survey questionnaire was thus divided into multiple sections spanning demographic group, socio-economic status, attitude towards mental health, and care-seeking behaviour/prevention, to survey and promote mental health literacy. Through the study, it was established that mental health literacy of tribal communities is mainly impacted by access to education, cultural differences, healthcare access, and economic deprivation. Further research must be conducted to evaluate how mental health literacy can be promoted through survey questionnaire and community media/radio within tribal Indian communities.

